# Effectiveness of a needs-tailored nurse-led recovery program for community-dwelling people with schizophrenia: a cluster-randomized controlled trial

**DOI:** 10.1186/s12912-024-01986-x

**Published:** 2024-05-16

**Authors:** Wen-I Liu, Wen-Ling Hsieh, Ching-Ting Lai, Chia-Chen Liu, Yueh-Ming Tai, Chieh-Yu Liu

**Affiliations:** 1 School of Nursing, National Taipei University of Nursing and Health Sciences, Taipei City, Taiwan; 2 Tri-Service General Hospital Beitou Branch, Taipei City, Taiwan; 3 Department of Health Care Management, National Taipei University of Nursing and Health Sciences, Taipei City, Taiwan

**Keywords:** Community psychiatric nurses, Needs-tailored, Recovery, Schizophrenia, Cluster-randomized controlled trial

## Abstract

**Background:**

Meeting people’s needs is positively correlated with their recovery. However, recovery services rarely include nurse-led programs tailored to the needs of these people. This study aimed to evaluate the effectiveness of a new needs-tailored recovery program by using a cluster-randomized controlled trial design.

**Methods:**

We conducted a parallel randomized controlled trial in two community psychiatric departments, employing nurse-level clustering for intervention delivery and selecting participants through convenience sampling. The participants were people diagnosed with schizophrenia that were receiving homecare services. The experimental group (*n* = 82) received needs-tailored recovery program for six months. The control group (*n* = 82) received traditional homecare. Data were collected at baseline, post-intervention, and the three-month follow-up (the study ran from February to December 2021). The outcomes were recovery, needs, hope, empowerment, psychotic symptoms, and medication adherence. We used repeated measures ANOVA tests to examine the effect of the group × time interaction.

**Results:**

The participants in the experimental group demonstrated statistically significant improvements in recovery, hope, and medication adherence compared to the control group, both immediately post-intervention and at the three-month follow-up. Moreover, they exhibited statistically significant reductions in needs compared to the control group at the three-month follow-up (*p* < .05). While the interaction effect for psychotic symptoms was not significant, the time effect was significant (*p* < .05). No significant interaction or time effect was observed for empowerment.

**Conclusion:**

The findings increase our understanding of recovery-oriented care that prioritizes therapeutic alliance, integrated needs assessment, individual goals, hope, and empowerment.

**Trial registration:**

The Clinicaltrials.gov identifier NCT05304780 retrospectively registered on 03/31/2022.

## Background

Recovery-oriented care has attracted considerable attention globally and is considered the goal of mental health care [1]. Personal recovery is a multifaceted concept and can be defined as both the personal process of living with serious mental illness and the results of mental health care [2, 3]. Some developed countries have established related programs and evaluated their effectiveness in improving recovery rates [4]. Meeting the needs of community-dwelling people with schizophrenia is positively correlated with their recovery [5, 6]. In a longitudinal study, it was demonstrated that systematically monitoring patients’ needs can improve their psychotic symptoms, thereby promoting their recovery. The degree to which patients’ needs are met is significantly and positively correlated with the extent of their recovery [6]. However, 25–50% of people with serious mental illness have unmet needs [7], which may exacerbate their psychotic symptoms and hinder their recovery [8].

In Taiwan, community psychiatric nurses play a vital role in delivering mental health care services to people with psychiatric disorders who reside in the community and experience residual symptoms, often without access to continuous treatment in mental health institutions. These services provided by community psychiatric nurses typically include medication treatment, health education, and family consultations, aiming to address the disease-oriented needs of people with psychiatric disorders. Despite the increasing demand for support in this population, existing care services have not adequately expanded to meet these needs. While temporary care gap solutions, such as community care quality promotion programs, have been implemented, formal, needs-tailored, and recovery-oriented home care services are notably absent. Moreover, the current community mental health care system primarily focuses on medical treatment and lacks an integrated care model that caters to individual needs. Consequently, there is an urgent imperative to develop integrated and needs-tailored recovery care services to bridge this gap and better support people with psychiatric disorders [9].

Based on a systematic review, the complex and multifaceted needs of community-dwelling people with schizophrenia are as follows: mental recovery, disease management, life management, crisis management, family support, social participation, and resource connection [10]. These services should be provided by multidisciplinary teams. Case management processes should provide integrated services that can effectively reduce the number of hospitalizations for community-dwelling patients, connect them with professional services, enhance the quality of care, and improve psychosocial outcomes [10–12]. Current recovery program providers are mostly psychologists, occupational therapists, and rehabilitation therapists, and nurse-led, recovery-oriented, and individualized care services are rare [2, 4]. Psychiatric homecare nurses, who make up the largest group of community-based care professionals, have the most direct contact with this population. Their unique role in providing integrated care for community-based personal recovery should be expanded and the effectiveness of person-centered, individualized recovery programs must be developed and assessed [13].

In many countries, there is currently a trend of mental health services shifting from the hospital setting to community-based care. Emphasizing person-centered and recovery-oriented values is increasingly becoming a core concept in psychiatric care [14, 15]. Psychiatric mental health nurses can work together with service users to support recovery processes [16]. However, the recovery services offered globally seldom comprise nurse-led, person-centered programs that are tailored to the needs of such people. Most Asian people with schizophrenia live at home in the community rather than in institutions [17]. As highlighted by the National Health Research Institute, the needs of people with schizophrenia in Taiwan have not been fully evaluated and community care services have been unable to respond to their needs for individualized care [13]. Consequently, the primary aim of this cluster-randomized controlled trial was to assess the effectiveness of a novel needs-tailored recovery program on various outcomes, including recovery, needs, hope, empowerment, medication adherence, and psychotic symptoms among community-dwelling people with schizophrenia. We hypothesized that participants receiving the needs-tailored recovery program, compared to those in the usual care control group, would demonstrate significantly enhanced levels of recovery, hope, empowerment, and medication adherence, alongside reduced levels of needs and psychotic symptoms. If successful, such a program would move community homecare services toward a forward-looking, evidence-based, and innovative model of care for community-dwelling people with schizophrenia.

As highlighted by the National Health Research Institute, the assessment of needs among people with schizophrenia in Taiwan remains incomplete, and existing community care services have struggled to meet their requirements for personalized care [13]. Consequently, the primary objective of this cluster-randomized controlled trial was to assess the impact of a novel needs-tailored recovery program on various outcomes, including recovery, needs, hope, empowerment, medication adherence, and psychotic symptoms, among people with schizophrenia residing in the community. Our hypothesis posited that participants receiving the needs-tailored recovery program, in contrast to those in the usual care control group, would exhibit significantly enhanced levels of recovery, hope, empowerment, and medication adherence, alongside reduced levels of needs and psychotic symptoms.

## Methods

### Design

This was a cluster-randomized controlled trial with convenience sampling. The study was conducted over two years in two phases—development and evaluation. In the first phase, from August 2019 to June 2020, we developed the program and conducted expert content validity tests based on a systematic review and the Delphi method. The current manuscript describes the second phase, which was conducted between February and December 2021 in Taiwan and includes an effectiveness evaluation. We first conducted a pilot study to refine the program, followed by a parallel cluster-randomized controlled trial with a repeated measures design to evaluate the effectiveness of the intervention. The trial was registered at NCT05304780. This study was grounded in the CONSORT checklist for reports of randomized trials.

### Participants

The participants were recruited from the community psychiatric departments of two research institutes in northern Taiwan. These institutes provide homecare for the largest number of people diagnosed with schizophrenia in Taipei City and New Taipei City. All research institutions are psychiatric teaching hospitals that share uniformity in terms of size, certification grade, competencies of community psychiatric nurses, and availability of psychiatric home care equipment. Each research institute typically employed six community psychiatric nurses. In total, approximately 1000 community-dwelling clients received care from the two research institutions, including around 600 diagnosed with schizophrenia, some of whom exhibit residual symptoms of psychosis.

People who met the following criteria were enrolled as study participants: (1) were living in the community and had a diagnosis of schizophrenia based on the *Diagnostic and Statistical Manual of Mental Disorders, Fifth Edition* (DSM-5); (2) were aged 20–64 years; and (3) were able to communicate in Mandarin or Taiwanese. We applied these criteria to reduce participant heterogeneity and any potential communication difficulties.

Potential participants were excluded if they (1) were living in a community institution such as a recovery home, community rehabilitation center, nursing home, or day hospital; or (2) had a neurocognitive disorder, substance abuse disorder, or comorbidity.

### Randomization

Given the primary nursing care model adopted in psychiatric home care, each community psychiatric nurse is responsible for providing home care within their assigned area. As a result, random assignment of patients was not feasible. Hence, this study employed nurse-level clustering for intervention delivery.

To ensure homogeneity in the education level and proficiency of the interventionists, as well as consistency in intervention delivery, selection criteria included holding a bachelor’s degree, possessing over five years of experience, and passing the community psychiatric mental health competency assessment.

To balance group sizes and ensure an adequate sample size across groups, a total of eight nurses, with four in each institution meeting the above criteria, were selected for this study. In each institution, two nurses were randomly selected as interventionists in the experimental group, while the other two were assigned to the control group. Random selection was performed using the RANDBETWEEN function in Microsoft Excel, and this process was conducted by a blinded allocator.

Each nurse screened and selected patients meeting the inclusion criteria for participation. Convenience sampling, based on patient availability or accessibility, streamlined data collection, saving time and costs. Nurses in the experimental group provided needs-tailored recovery interventions, while those in the control group delivered traditional home care.

### Blinding

The interventionists were aware of group allocation because they had to be given specific training and offer the intervention, but the participants were blinded to their group allocation. Both groups received home visits by the psychiatric homecare nurses, who were asked not to reveal their group assignment to the participants. The data were collected by two trained research assistants who were blinded to intervention to reduce potential bias in the data collection.

### Sample size

As a post hoc validation procedure, we used G*Power version 3.10 to estimate the power of the study given our sample size and analysis methods. We employed repeated-measures ANOVA to compare changes between the two study groups at three time points. The statistical test utilized was the within-between interaction, assuming an effect size of 0.11 [18], with 80% power and a significance level of 5%. Prior to the trial, the estimated total sample size was 136, accounting for an anticipated 20% loss rate, resulting in an expected total of 164 participants.

### Intervention: the needs-tailored recovery program

**Fig. 1:**
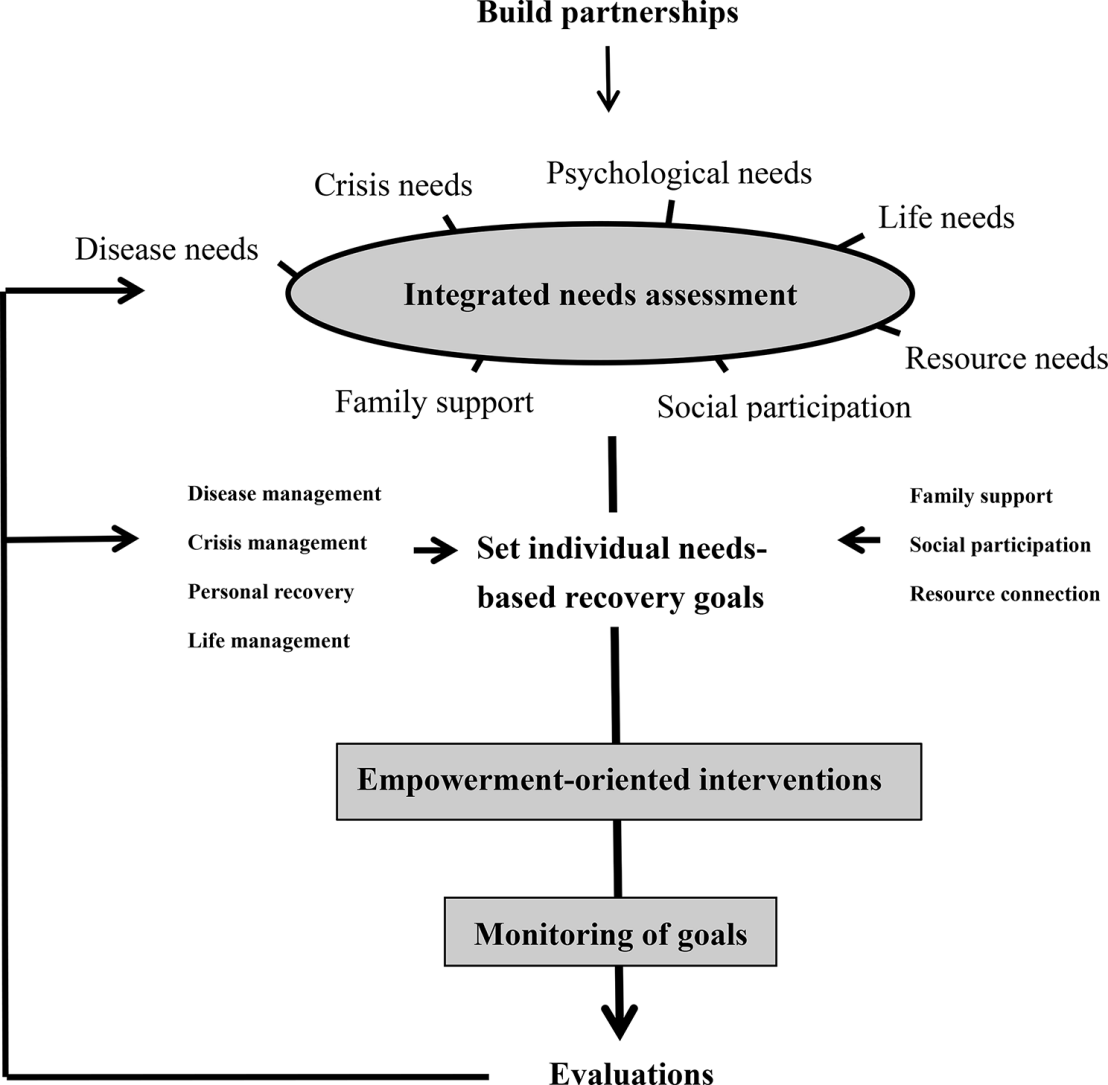
The needs-tailored nurse-led recovery program

We conducted a systematic review, extracted the essential components for the recovery program, and developed an intervention manual with standardized workflows and service content. The content validity index of the program was assessed by eight clinical experts and scholars in community psychiatric mental health. The feasibility, efficacy, and cost-effectiveness of the intervention were considered adequate, based on its content validity index of 0.97. The recovery program integrated the following evidence-based essential components that we had identified as important for community-dwelling people with schizophrenia: care needs, empowerment, and medication adherence, as well as hope [3, 19]. The intervention frequency comprised a minimum of one home visit every two weeks, spanning a duration of six months. The intervention process consisted of five steps (refer to Fig. 1), encompassing a total of 12 home visits, each lasting approximately 50 min. Initially, emphasis was placed on building relationships, followed by two visits dedicated to integrated needs assessment. Subsequently, eight visits focused on providing empowerment-oriented interventions and monitoring goals, culminating in a final visit for evaluations.

This intervention process adhered to a standard manual, with each step recorded using a checklist to ensure consistency. Interventionists conducted discussion and training meetings based on the standard manual and underwent consistency training before commencing the intervention. Further details are provided below:

*Step 1: Build partnerships*: create a partnering environment, develop self-awareness, show empathy and sincerity, and provide support.

*Step 2: Conduct integrated needs assessment*: use the needs assessment scale for community-dwelling people diagnosed with schizophrenia to identify their needs and priorities. The development of this scale was primarily informed by a systematic literature review and referenced the Camberwell Assessment of Need (CAN), a comprehensive evaluation of patients’ needs [20]. This tool comprises two parts: the first part is a score sheet ranging from 0 to 2 points, while the second part involves qualitative records obtained through visit interviews and interactions.

*Step 3: Set needs-based recovery goals and provide empowerment-oriented interventions as follows*:

1. Disease management: improve disease-response capacity, strengthen medication motivation, promote self-management of medications, and encourage alertness for and prevention of recurrence.
2. Crisis management: respond to stress, know how to deal with an immediate crisis affecting the client or their family, and activate crisis management through healthcare professionals.
3. Personal recovery: recognize the aspects of personal recovery, use self-empowerment, and overcome self-stigma.
4. Life management: promote motivation, planning, and implementation of life management and strengthen support.
5. Family support: promote consistent communication within the family, meeting family responsibilities, and family-based problem solving.
6. Social participation: improve social skills, provide peer support, and maintain appropriate social activities.
7. Resource connection: provide resource information, enhance motivation to use resources, and encourage the development of knowledge of and the ability to use resources.

*Step 4: Conduct continuous monitoring of goals.*

*Step 5: Conduct effectiveness evaluations*: treat recovery as the primary outcome and the other elements (needs, hope, empowerment, medication adherence, and psychotic symptoms) as the secondary outcome measures.

### Traditional homecare as usual care

Traditional homecare, serving as the usual care in this study, involved community psychiatric nurses and doctors conducting home visits for psychiatric patients. These visits encompassed the provision of medication treatment, health education, and family support to assist patients with living in the community. The frequency and duration of the visits were consistent with those of the experimental group, occurring every two weeks and lasting 50 min each, over a period of six months.

### Data collection

Two nursing research assistants who were experienced in psychiatric care collected the data. They measured the effectiveness of the program by using reliable, validated questionnaires at the beginning and end of the intervention period and at a follow-up session three months later. The following basic demographic data were collected: gender, age, education level, marital status, living status, employment status, religion, economic status, duration of receiving homecare, total number of psychiatric hospitalizations, and number of such hospitalizations during the past year. Guided by the questionnaire, assessments of the participant’s personal recovery, empowerment, needs, hope, psychotic symptoms, and medication adherence were collected. The psychotic symptoms were assessed by the interviewers and the participants answered the remaining questions themselves. If the participants did not understand any of the questions, the interviewer provided assistance and instructions.

### Outcome measurements

This study included seven outcome measures. The primary outcome was recovery, while the secondary outcomes consisted of needs, hope, empowerment, medication adherence, and psychotic symptoms. Assessments were conducted at baseline, post-intervention, and three-month follow-up. Nurses administered assessments for needs and psychotic symptoms, while participants self-assessed the other outcomes. Further details are provided below:

#### Recovery

We used the Questionnaire about the Process of Recovery, developed and validated by Neil et al. (2009) [21], to evaluate the participants’ recovery. This instrument has been widely used internationally and was translated by Chien and Chan into Mandarin in 2013. The higher the score, the greater the recovery of the respondent. The total variance explained was > 48%, Cronbach’s α was 0.77–0.94, and the two-week test–retest reliability was 0.77–0.87 [22].

#### Needs

The development of this needs scale was based mainly on a systematic literature review of studies [10] and referencing the Camberwell Assessment of Need [23–25]. The associated questionnaire has a total of 22 questions, with each question scored from 0 to 2 points. The higher the score, the higher the level of needs. Cronbach’s α for this questionnaire was 0.81, and the total variance explained in the factor analysis was 61.23% [20].

#### Hope

We used the Herth Hope Index, which comprises 12 questions, to assess hope. These questions are answered using a four-point Likert scale, and the higher the score, the higher the level of hope [26]. The index is the most widely translated and thoroughly psychometrically tested tool in languages other than English [27]. Chan et al. (2011) translated this scale into Chinese and found that the reliability and validity of this version were good [28].

#### Empowerment

We used the Empowerment Scale developed by Rogers et al. (1997) [29]. We used the Mandarin version, which was translated by our research team and verified using people diagnosed as having schizophrenia. The content validity index was 0.88. The total variance explained was 59%. Cronbach’s α was 0.87 [30].

#### Medication adherence

We used the Medication Adherence Rating Scale developed by Thompson et al. (2000) [31]. The associated questionnaire consists of 10 questions and has a total score of 0–10 points. The higher the score, the better the adherence to medication. The questionnaire was translated into Mandarin by Kao and Liu (2010) [32]. Cronbach’s α for this questionnaire was 0.72, with a two-week test–retest reliability of 0.80 and 49.7% of the total variance explained [32].

#### Psychotic symptoms

We used the Brief Psychiatric Rating Scale developed by Overall and Gorham (1962) to evaluate the participants’ psychotic symptoms [33]. The score for each of the 16 questions is 0–7 points, and the higher the score, the more obvious the psychotic symptoms. Chang et al. (1986) translated the scale into Mandarin and found that the reliability and validity of the scale were good [34].

### Data analysis

Loss of participants occurred primarily due to reasons such as rehospitalization and declining to respond. Despite the occurrence of some loss, it remained within an acceptable range and did not fall below the estimated total sample size of 136, ensuring a statistical power of over 0.8. Therefore, we chose not to utilize imputation methods to address missing data. Instead, we conducted a per-protocol (PP) analysis. We used IBM SPSS 25.0 (IBM, Armonk, NY, US) for data entry and analysis. We first conducted a descriptive analysis of the data using averages, standard deviations, numbers, and percentages. We then compared the homogeneity of the demographics and the pretest outcome measures between the groups using *t*-tests and chi-squared tests. We employed repeated-measures ANOVAs to examine differences between the two groups, focusing on group-by-time interactions for each outcome variable. We first tested the assumptions for the ANOVA (independence, normality, and sphericity), and where the data did not satisfy the requirement of sphericity, we applied the Huynh–Feldt correction.

## Results

### Demographics, mental health status, and homogeneity tests of the two groups

**Fig. 2:**
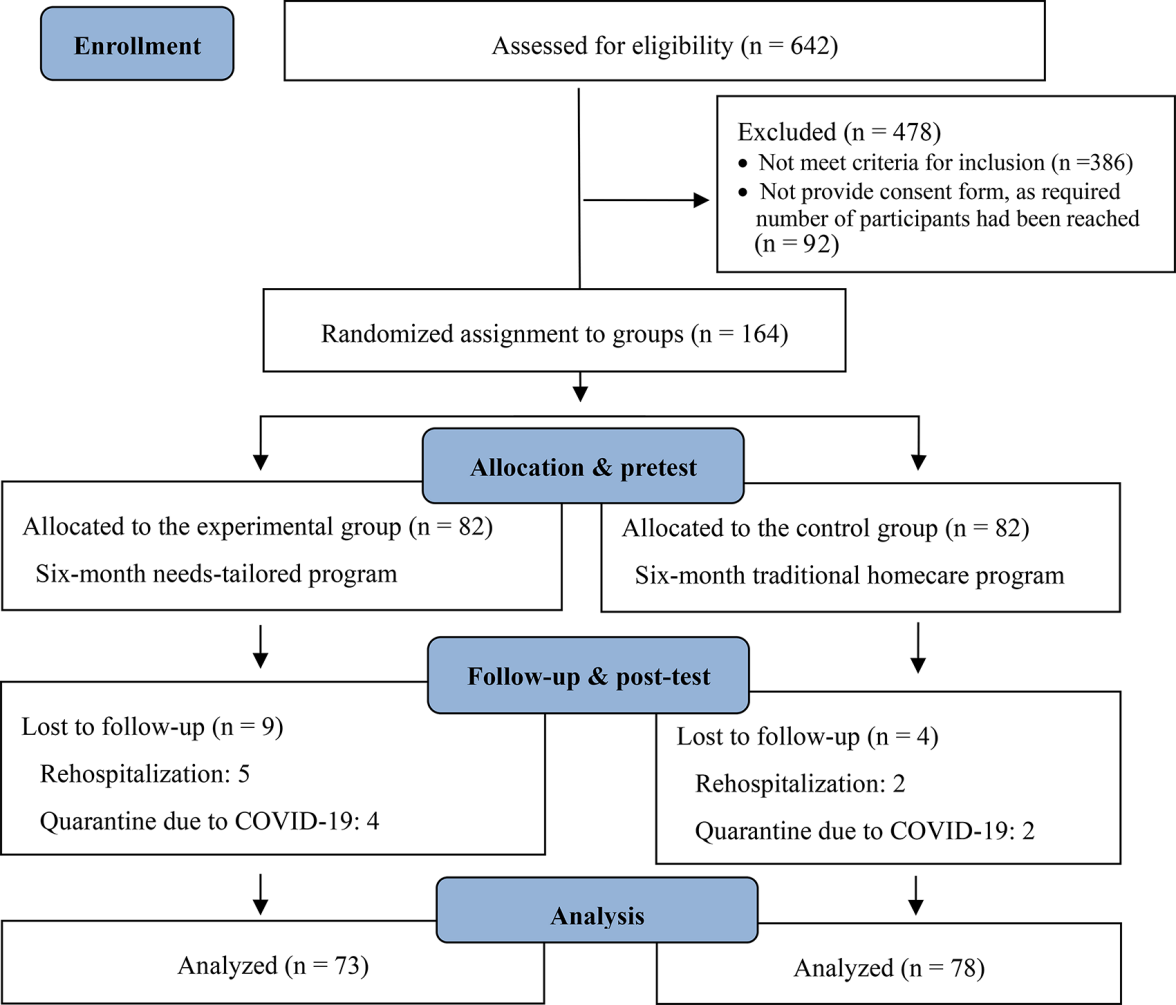
Flow diagram for participant recruitment and retention

Each research institution had four community psychiatric nurses, who were randomly selected as either experimental group interventionists or control group providers, with each nurse responsible for approximately 80 cases. Under the eight community psychiatric nurses, a total of 642 patients who received psychiatric home care were initially included to assess for eligibility. However, 478 patients either did not meet the inclusion criteria or did not provide the research consent form, as the required number of participants had already been reached. Consequently, a total of 164 participants were included in this study (Fig. 2). Each group contained 82 participants. During the study, 13 participants withdrew due to hospitalization or quarantine related to COVID-19, resulting in a subject loss rate of 7.9%. No statistically significant differences were observed between participants who completed the study and dropouts regarding demographics, clinical variables, and outcome variables (*p* > .05). The analysis comprised 151 participants, with post-hoc analysis revealing a statistical power of 0.85.

The study had more male participants (58.9%) than female. The mean age of these participants was 49.53 ± 9.27 years. The majority of the participants were high school or vocational training graduates (43.0%) who were living with family members (90.7%), unemployed (76.8%), Buddhist (42.4%), and belonged to a low-income household (52.3%). Most participants (66.9%) had received homecare for more than two years. The average total number of psychiatric hospitalizations per participant was 3.59 ± 3.95. The average number of psychiatric hospitalizations per participant in the previous year was 0.15 ± 0.44 (Table 1).

**Table 1:** Demographic data homogeneity for the two participant groups

	Overall (*N* = 151)	Experimental (*n* = 73)	Control (*n* = 78)	*t*^a^ /χ^2 b^	*p*
	*n* (%)/mean ± SD	*n* (%)/mean ± SD			
Gender				0.43 ^b^	0.514
Male	89 (58.9)	45 (61.6)	44 (56.4)		
Female	62 (41.1)	28 (38.4)	34 (43.6)		
Age	49.53 ± 9.27	48.35 ± 9.84	50.63 ± 8.63	−1.51 ^a^	0.132
Education level				2.64 ^b^	0.267
Middle school and below	62 (41.1)	31 (42.5)	31 (39.7)		
High school or vocational school	65 (43.0)	34 (46.5)	31 (39.8)		
College and above	24 (15.9)	8 (11.0)	16 (20.5)		
Marital status				0.42 ^b^	0.813
Single	106 (70.2)	50 (68.5)	56 (71.8)		
Married	25 (16.6)	12 (16.4)	13 (16.7)		
Divorced/widowed	20 (13.2)	11 (15.1)	9 (11.5)		
Living status				0.02 ^b^	0.896
Living alone	14 (9.3)	7 (9.6)	7 (9.0)		
Living with family	137 (90.7)	66 (90.4)	71 (91.0)		
Employed				0.01 ^b^	0.976
No	116 (76.8)	56 (76.7)	60 (76.9)		
Yes	35 (23.2)	17 (23.3)	18 (23.1)		
Religion				0.38 ^b^	0.945
None	32 (21.2)	17 (23.3)	15 (19.2)		
Buddhist	64 (42.4)	30 (41.1)	34 (43.6)		
Taoist	32 (21.2)	15 (20.5)	17 (21.8)		
Christian	23 (15.2)	11 (15.1)	12 (15.4)		
Economic status				0.07 ^b^	0.792
Middle income	72 (47.7)	34 (46.6)	38 (48.7)		
Low income	79 (52.3)	39 (53.4)	40 (51.3)		
Number of years of homecare received				4.58 ^b^	0.101
< 1 year	14 (9.3)	6 (8.2)	8 (10.3)		
1–2 years	36 (23.8)	23 (31.5)	13 (16.6)		
≥ 2 years	101 (66.9)	44 (60.3)	57 (73.1)		
Total number of psychiatric hospitalizations	3.59 ± 3.95	3.79 ± 4.30	3.40 ± 3.59	0.62 ^a^	0.538
Number of psychiatric hospitalizations within the previous year	0.15 ± 0.44	0.19 ± 0.52	0.10 ± 0.35	1.24 ^a^	0.218

*Note*^a^*t*-test; ^b^ chi-squared test

At baseline, there were no statistically significant differences in demographics, clinical variables, and outcome variables between the experimental and control groups (Tables 1 and 2).

**Table 2:** Effectiveness of the needs-tailored nurse-led recovery program

	Descriptive and homogeneity tests				Group effect		Time effect		Group*Time	
	Experimental	Control	*t*	*p*	*F*	*p*	*F*	*p*	*F*	*p*
**Recovery**					**1.71**	**0.193**	**4.42**	**0.013**	**5.46**	**0.005**
Pretest	55.95 ± 10.46	56.72 ± 9.23	−0.48	0.631						
Post-test	57.40 ± 12.99	54.04 ± 11.36					0.62	0.432	7.04	0.009
Follow-up	59.92 ± 14.35	55.99 ± 10.58					3.96	0.048	8.34	0.004
**Empowerment**					**0.34**	**0.561**	**0.13**	**0.857**	**0.91**	**0.397**
Pretest	33.49 ± 4.23	33.36 ± 3.24	0.20	0.843						
Post-test	33.31 ± 4.67	33.23 ± 3.53					0.33	0.564	0.10	0.922
Follow-up	33.76 ± 5.07	32.96 ± 3.74					0.29	0.864	0.94	0.334
**Need**					**1.44**	**0.232**	**88.66**	**< 0.001**	**13.91**	**< 0.001**
Pretest	10.78 ± 4.84	9.71 ± 5.97	1.21	0.228						
Post-test	6.52 ± 3.50	6.88 ± 3.97					88.43	< 0.001	3.66	0.058
Follow-up	3.95 ± 3.35	6.81 ± 4.95					137.77	< 0.001	22.56	< 0.001
**Hope**					**2.34**	**0.128**	**5.22**	**0.008**	**4.22**	**0.019**
Pretest	32.30 ± 4.69	32.36 ± 3.67	−0.08	0.933						
Post-test	33.66 ± 5.33	32.40 ± 4.03					4.95	0.028	4.42	0.037
Follow-up	34.32 ± 6.00	32.47 ± 4.32					7.41	0.007	5.89	0.016
**Medication adherence**					**8.20**	**0.005**	**5.65**	**0.006**	**4.73**	**0.013**
Pretest	6.32 ± 2.50	5.92 ± 2.88	0.89	0.375						
Post-test	7.26 ± 2.39	5.73 ± 2.83					3.15	0.078	7.20	0.008
Follow-up	7.38 ± 2.20	6.14 ± 2.92					9.47	0.002	4.14	0.044
**Psychotic symptoms**					**2.98**	**0.086**	**4.10**	**0.035**	**0.43**	**0.555**
Pretest	9.66 ± 8.09	12.51 ± 13.81	−1.56	0.121						
Post-test	8.93 ± 7.72	10.46 ± 8.34					2.38	0.125	0.54	0.463
Follow-up	8.07 ± 6.76	9.87 ± 7.53					5.91	0.016	0.37	0.546

### Effect on recovery, needs, hope, empowerment, medication adherence, and psychotic symptoms

Participants in the experimental group demonstrated statistically significant improvements in recovery compared to the control group, both immediately post-intervention (*F = 7.04, p* = .009) and at the 3-month follow-up (*F = 8.34, p* = .004). Furthermore, at the 3-month follow-up, the experimental group exhibited statistically significant decreases in needs compared to the control group *(F = 22.56, p* < .001).

Additionally, participants in the experimental group showed statistically significant improvements in hope compared to the control group, both immediately post-intervention (*F = 4.22, p* = .037) and at the 3-month follow-up (F = 5.89, *p* = .016). Finally, participants in the experimental group displayed statistically significant improvements in medication adherence compared to the control group, both immediately post-intervention (*F = 7.20, p* = .008) and at the 3-month follow-up (*F = 4.14, p* = .044), as evidenced by repeated-measures ANOVA.

**Fig. 3:**
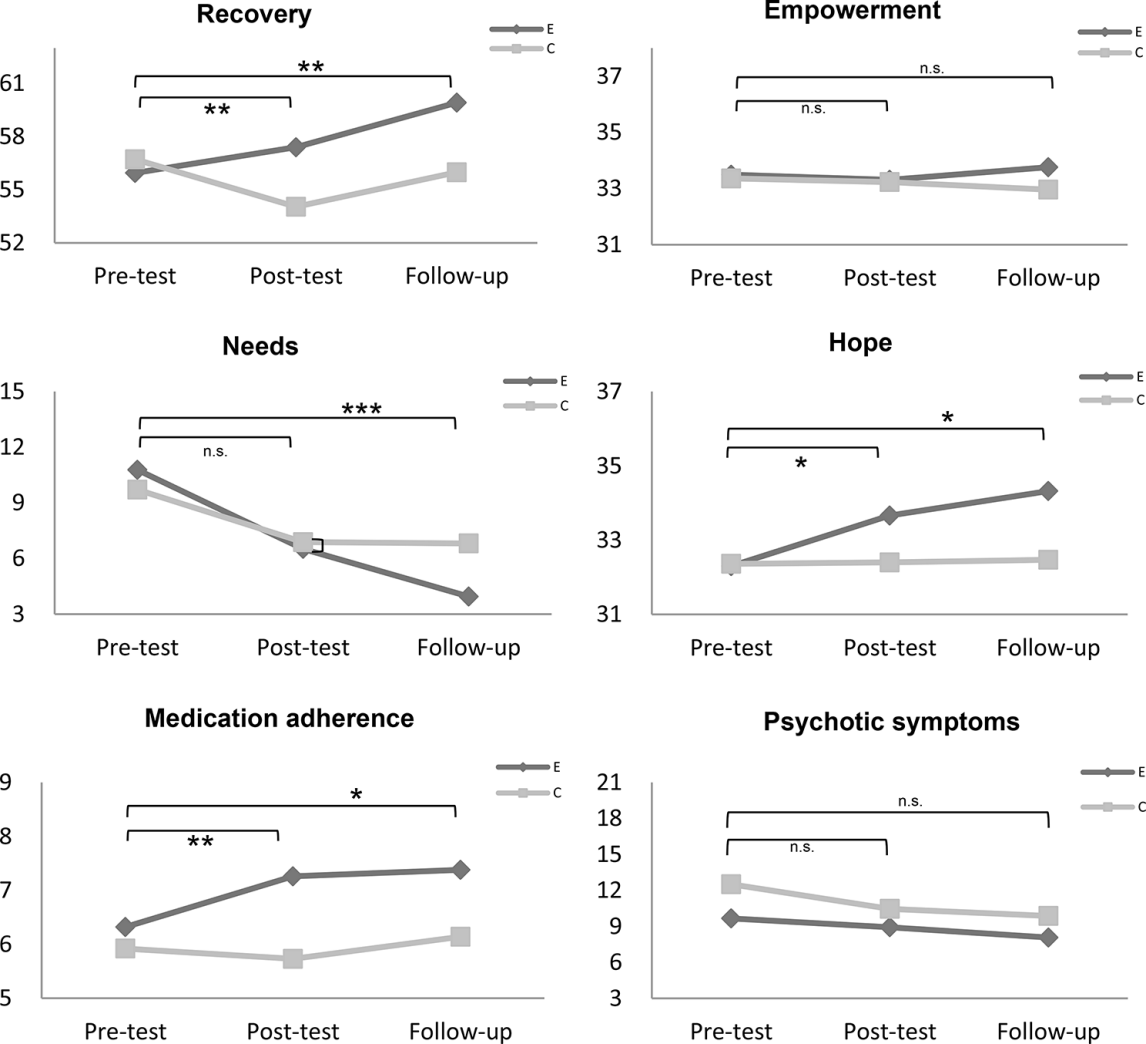
Group differences in outcomes over time. E = experimental group; C = control group; * = *p*-values < 0.05 of interaction effects; ** = *p*-values < 0.01 of interaction effects; *** = *p*-values < 0.001 of interaction effects

Although there were no statistically significant group differences in psychotic symptoms, there was a statistically significant time effect (*F* = 4.10, *p* = .035). There were no statistically significant group or time effects on empowerment (Table 2; Fig. 3).

## Discussion

We found that a needs-tailored recovery program was more effective than traditional homecare for improving recovery, needs, hope, and medication adherence in people with schizophrenia, and that its effects were sustained. This finding aligns with systematic literature, indicating that adequate hope, family, and social support are key facilitators of recovery. This finding aligns with previous literature, which suggests that factors such as adequate hope, family support, social support, and reduced needs can contribute to facilitating recovery [9, 35]. In a recovery program, the goal of care must align with and be tailored to the individual patients’ needs [8, 36]. Our results support previous research that shows that people with schizophrenia have complex and multifaceted needs, which are influenced by a range of clinical, psychological, social, economic, and occupational factors [37]. Our needs-tailored, person-centered recovery program takes into account the aforementioned aspects and has proven to be effective in supporting patient recovery. Additionally, existing literature suggests that recovery is not only an outcome but also a continuous process [38]. Notably, our program demonstrated significant post-intervention effects, outperforming traditional homecare at the three-month follow-up. This underscores the program’s ability to support patients throughout their ongoing recovery journey and highlights its value as an intervention. Our needs-tailored, person-centered recovery program considers the abovementioned aspects and is effective in supporting patient recovery.

This confirms that this evidence-based program, which was designed based on quantitative literature [3, 19, 39, 40] and whose effectiveness was evaluated using a randomized control trial design, is beneficial both in terms of its theoretical applications and for practical clinical work. It would be able to meet the disease-related, psychological, and social participation of more than 50% of existing people diagnosed as having schizophrenia and provide service strategies that respond to the needs assessed in this study. Schizophrenia is a substantial burden on the global community, and as an economic burden accounts for 0.02–1.65% of total production value. Given the limited available medical resources and other competing and urgent demands, effectively promoting patient recovery could reduce the burden placed on limited healthcare workforces [41]. This innovative recovery program is person-centered and individualized. It has good expert validity and empirical evidence supports its effectiveness. It could be used to guide future mental health policies and practices and serve as a leading nurse-led model for care practices.

One possible explanation for the program’s lack of significant impact on empowerment could be the cultural mismatch between the empowerment concepts utilized. The Chinese version of the empowerment scale, translated from Roger’s Empowerment Scale, may be more aligned with Western culture. However, literature suggests that in some Asian populations, empowerment may be perceived differently, with a focus on maintaining a satisfactory quality of life by remaining passive and minimizing stressors [35]. Additionally, the participants used to validate Roger’s Empowerment Scale or the Chinese version of the Empowerment Scale may have been individuals with stable chronic psychotic disorders in a community institution setting or chronic ward, whose characteristics may not fully align with those of the participants in our study [29, 30]. Therefore, it is recommended to utilize measurement tools that are more aligned with Eastern culture when assessing empowerment among homecare psychiatric patients.

We also did not observe any statistically significant differences in psychotic symptoms between the two groups, although these symptoms were reduced over time in both. A possible reason for this is that since the participants were community-dwelling people with stable psychotic symptoms not requiring hospitalization [42], they were in the nonacute phase of the disease and had fewer symptoms anyway, although they did still require continuous care. The other possibility is that the usual homecare is already sufficient to provide statistically significant improvements in psychiatric symptoms [43]. Further, the nurses who provided the services to both groups had passed the competence assessment and were qualified to provide community mental healthcare. Consequently, both groups received high-quality care and achieved positive results [44]. The fact that the study used homecare provided by nurses also revealed the unique role of nurses in promoting clients’ medication adherence, thereby reducing disease recurrence and rehospitalization and enabling them to live a stable life in the community [45, 46].

Adherence to medication involves multiple complex behaviors. In the future, continuous development and verification are required to design and apply empowerment-oriented, theory-based programs that combine diversified and empirical strategies to strengthen the motivation and attitudes toward medication adherence of people with schizophrenia [39]. In addition, ongoing evaluations may be necessary to track how effective these programs are in the long term.

## Limitations

Although we used random assignment and research instruments with good reliability and validity, the study nevertheless had several limitations. First, we used convenience sampling, voluntary participation, and conducted the study in northern Taiwan. These aspects may be associated with a risk of sampling bias and the samples may not be representative of the wider population. However, we did recruit participants from two different institutes to reduce the threat to external validity. Further, the participants were all home-based clients, and the service providers were asked not to exchange information during the intervention training, so the risk of cross-contamination between clients and service providers was low.

Finally, we respected the participants’ right to withdraw from the study and were therefore unable to collect post-test data from several of them. We did not conduct an intention-to-treat analysis. However, both the participants and the data collectors were blinded to group assignment to maintain construct validity.

## Conclusions

We developed an individualized recovery program in the form of a nurse-led homecare intervention that was tailored to the needs of community-dwelling people with schizophrenia. Compared with those who received the traditional homecare services, the recovery, hope, and medication adherence scores of the participants who received the experimental intervention were significantly improved. Additionally, they showed statistically significant decreases in needs compared to the control group.

This intervention is centered on addressing the diverse needs of people diagnosed with schizophrenia to promote recovery. It deviates from past disease-oriented care approaches by emphasizing the necessity of transitioning towards a recovery-oriented care model in the future. By focusing on holistic and individualized support, the intervention aims to empower patients and enhance their overall well-being, thereby facilitating long-term recovery outcomes.

Our findings contribute to the understanding of effective recovery-oriented care practices, which prioritize elements such as therapeutic alliance, integrated needs assessment, individual goal-setting, hope, and empowerment. This paradigm-shifting nurse-led program should be embraced as an innovative approach to community mental health care, serving as a practical guide for the care of people with schizophrenia residing in the community.

## Data Availability

The research data cannot be shared openly due to the necessity of safeguarding the privacy of individuals with schizophrenia and adhering to the regulations outlined in our Institutional Review Board’s agreement.
